# Nociceptive pain in late-onset Pompe disease: prevalence, distribution, and clinical correlates

**DOI:** 10.1007/s00415-026-14071-x

**Published:** 2026-08-25

**Authors:** Stephan Wenninger, Marcela Arndt, Daniel H. Mendelsohn, Corinna Wirner-Piotrowski, Marko Mijic, Natalia Garcia-Angarita, Kristina Gutschmidt, Christoph Schmidt, Lisa Winkler, Dieter Gläser, Karl Christian Knop, Matthias Spranger, Frank Klawonn, Lea Eileen Brauner, Benedikt Schoser

**Affiliations:** 1 Friedrich Baur Institute at the Department of Neurology, LMU University Hospital, LMU Medizin, Ludwig-Maximilians-Universität München, Ziemssenstr. 1, 80336 Munich, Germany; 2 MVZ genetikum GmbH, Wegenerstr. 15, 89231 Neu-Ulm, Germany; 3 Neurologie Neuer Wall, Neuer Wall 19, 20354 Hamburg, Germany; 4 Neuropsychiatricum, Osterstraße 1a, 28199 Bremen, Germany; 5 Institute for Information Engineering, Ostfalia University, Am Exer 2, 38302 Wolfenbuettel, Germany

**Keywords:** Pain, Nociceptive, LOPD, Muskuloskelettal, FSHD, IBM

## Abstract

**Background:**

Nociceptive pain is often recognized as a relevant symptom in late-onset Pompe disease (LOPD), yet its prevalence, distribution, and underlying determinants remain insufficiently characterized. We aimed to systematically assess nociceptive pain in LOPD and to explore its relationship with muscle function, structural muscle alterations, and potential modifiers.

**Methods:**

In this multicenter cross-sectional study, 42 patients with LOPD and 61 disease controls (IBM, FSHD, SMA3) were evaluated. Pain was assessed using validated questionnaires and pain drawings. Motor function tests, pressure pain thresholds, muscle ultrasound, laboratory parameters, and genetic polymorphisms (ACE, ACTN3) were obtained. Associations between pain and clinical, structural, and biological variables were analyzed.

**Results:**

Chronic nociceptive musculoskeletal pain was highly prevalent, particularly in LOPD (83.3%) compared to disease controls (*p*=0.005). However, pain reported on the assessment day was significantly lower in LOPD (30.9%, *p*<0.001) and SMA3 (25%, *p*=0.016). No significant associations were found between pain and muscle strength, motor function tests, ultrasound-detected muscle alterations, ACTN3 and ACE, and laboratory parameters.

**Conclusion:**

Musculoskeletal pain is highly prevalent in LOPD but remains dissociated from objective measures of motor impairment, structural changes of muscle tissue, or genetic modifiers. The significant discrepancy between chronic pain history and acute pain reporting particularly in LOPD and SMA3 suggests that pain represents an independent clinical domain that is not fully captured by single-point assessments. Clinical management should focus on individualized pain assessment, as standard functional tests and snapshot pain assessments do not adequately reflect the patient’s pain burden.

**Supplementary Information:**

The online version contains supplementary material available at https://doi.org/10.1007/s00415-026-14071-x.

## Introduction

Pompe disease (glycogen storage disorder type II) is a rare autosomal recessive disorder affecting some 5000 to 10,000 individuals globally caused by acid α-glucosidase deficiency in the GAA gene, leading to lysosomal glycogen accumulation and progressive multisystem involvement. Late-onset Pompe disease (LOPD) is clinically characterized by slowly progressive proximal and axial myopathy with variable respiratory impairment and marked clinical heterogeneity that is not fully explained by GAA genotype alone [10, 14, 20].

Beyond motor impairment, musculoskeletal pain is increasingly recognized as a relevant but underreported symptom in LOPD. It may occur early in the disease course and becomes more prevalent with disease progression, substantially impairing quality of life. Despite enzyme replacement therapy (ERT), which can stabilize or slow motor decline, pain frequently persists or worsens, indicating that its underlying mechanisms are insufficiently understood [12, 16, 21, 35]. In particular, it remains unclear whether pain in LOPD is directly related to measurable disease severity, such as muscle weakness or structural muscle alterations, or whether additional factors, including potential modulators, contribute to its manifestation. Previous studies investigating pain in Pompe disease are scarce and largely limited to questionnaire-based assessments and only one used objective clinical assessments, providing little insight into its relationship with objective measures of muscle function or structural muscle changes [12, 21]. Genetic polymorphisms affecting muscle structure and function, such as the angiotensin-converting enzyme (ACE) insertion/deletion polymorphism and the ACTN3 R577X variant, have been proposed as modifiers of muscle phenotype and disease severity in neuromuscular disorders [3, 6, 10, 18, 19, 22]. While the ACE insertion/deletion (I/D) polymorphism is associated with variations in muscle metabolic efficiency and fiber distribution, the ACTN3 R577X variant dictates the presence or absence of alpha-actinin-3 in fast-twitch fibers. Deficiency in this protein (the XX genotype) has been linked to altered muscle structural resilience and shifted metabolic profiles. While it is well recognized that these variants are not disease-causing, it has been postulated that they may influence muscle performance, susceptibility to structural damage, and possibly the perception of pain [3, 6, 10]. In addition to genetic factors, biochemical markers such as Vitamin D have been widely established as modulators of muscle function and nociceptive pain. Vitamin D deficiency is frequently linked to myalgia and increased pain sensitivity, yet its specific impact on the pain profile in LOPD has not been systematically investigated [11, 15, 28, 37]. However, the role of these genetic and biochemical factors in modulating pain in LOPD remains poorly understood.

Taken together, there is a relevant gap in understanding the prevalence, distribution, and determinants of musculoskeletal pain in LOPD, particularly with respect to its relationship to objective measures of muscle function, structural muscle integrity, and potential biological modifiers.

The primary aim of this study was to systematically characterize the prevalence, severity, and spatial distribution of nociceptive musculoskeletal pain in adults with LOPD. Secondary objectives were to investigate associations between pain and muscle function, pressure pain sensitivity, structural muscle alterations assessed by ultrasound, as well as biochemical and genetic factors. To contextualize these findings, patients with other slowly progressive neuromuscular diseases (IBM, FSHD, and SMA3) and sharing a phenotype of slowly progressive, predominantly proximal and axial muscle weakness, but different in their underlying disease mechanisms, were included as disease controls.

## Methods

This nationwide, exploratory cross-sectional study was conducted at primary neuromuscular centers in Germany (Munich, Hamburg, Bremen). Adults (≥ 18 years) with genetically confirmed late-onset Pompe disease (LOPD) were eligible. Disease controls included patients with inclusion body myositis (IBM, confirmed by muscle biopsy), facioscapulohumeral muscular dystrophy (FSHD, genetically confirmed), and spinal muscular atrophy type 3 (SMA3, genetically confirmed), selected due to comparable patterns of proximal muscle involvement but different pathophysiology (muscular dystrophy, degenerative myopathy, neurogenic atrophy). Key exclusion criteria were current participation in interventional trials and clinically relevant depression, defined as a Beck Depression Inventory Fast Screen (BDI-FS) score ≥4 as well as continuous ventilation. As pain assessment focused on nociceptive musculoskeletal pain, participants with neuropathic pain and other extra-muscular pain entities in their current symptoms or medical history were not included in the primary analysis. Missing data were not imputed; analyses were performed on available cases. The study was registered at ClinicalTrials.gov (NCT05272969). The study protocol was approved by the Ethics Committee of the Faculty of Medicine, Ludwig Maximilian University (LMU) Munich, Germany (approval number 20–0977), and was conducted in accordance with the Declaration of Helsinki and applicable local regulations. All participants provided written informed consent prior to study participation.

### Clinical and functional assessment

Muscle strength was assessed using the Medical Research Council (MRC) scale. Functional endurance was evaluated using the six-minute walk test (6MWT) [1, 7, 9].

### Pressure pain sensitivity

Pressure pain threshold (PPT) was measured using pressure algometry at predefined muscle sites (trapezius, deltoid, supraspinatus, rectus femoris, and tibialis anterior). The mean of three measurements per site was recorded as kg/cm^2^ and used for analysis [8, 23].

### Muscle imaging

Muscle ultrasound was performed to assess structural muscle alterations, including muscle thickness and echogenicity, using the Heckmatt grading scale 1–4 across proximal, axial, and limb muscles [13, 24, 25, 33]. Although originally developed for dystrophic muscle disease, the Heckmatt score is a robust semiquantitative marker of muscle echogenicity and can also be applied in neurogenic and other neuromuscular disorders, including SMA and IBM [2, 26, 34, 36].

### Laboratory and genetic analyses

Blood samples were analyzed for creatine kinase, vitamin D, calcium, phosphate, and magnesium levels. Genotyping for ACE (I/D) and ACTN3 (R577X) polymorphisms was performed. Genomic DNA was isolated from peripheral blood samples using a standard extraction kit according to the manufacturer’s instructions (Chemagic DNA Blood Kit H24 (Perkin Elmer, Waltham, MA, USA), Flexigene DNA Kit (Qiagen, Hilden, Germany)). Exome sequencing based on the Twist Human Comprehensive Exome (Twist Bioscience, San Francisco, CA, USA) was performed. As a first step, genotyping for the ACE (I/D, rs4646994) and ACTN3 (R577X, rs1815739) polymorphisms was carried out using the megSAP pipeline (https://github.com/imgag/megSAP) followed by visual inspection with the Integrated Genomics Viewer [29].

### Patient-reported outcomes

Participants completed validated questionnaires assessing pain, function, and fatigue, including the Brief Pain Inventory (BPI), Fatigue Severity Scale (FSS), Rasch-built Pompe-specific Activity scale (RPAct), and the German Pain Questionnaire [4, 5, 17, 27, 31, 32]. Pain distribution was captured using body maps and digitally analyzed in Python to generate spatial pain patterns. Data quality checks and cleaning were performed prior to analysis to ensure consistent, valid records. The Pain drawings were available as binary image data with 850×600 pixel values. For spatial characterization, pixel-wise prevalence (“mean heat-maps”) was computed as the average value of each pixel across all binary drawings. Extra-muscular pain types (e.g., joint pain or neuropathic pain) were excluded.

### Outcomes

The primary outcome was the prevalence, severity, and spatial distribution of musculoskeletal pain in LOPD compared with disease controls. Secondary outcomes included associations between pain and muscle function, pressure pain sensitivity, muscle ultrasound findings, patient-reported outcomes, and biochemical and genetic variables.

### Statistical analysis

Given the exploratory design in rare diseases, analyses were primarily descriptive and inferential analyses should be interpreted as exploratory and hypothesis-generating. Continuous variables are reported as mean (SD) or median (IQR), as appropriate. Group comparisons were performed using one-way ANOVA or Kruskal–Wallis tests (depending on distribution), with Bonferroni-adjusted post hoc analyses where applicable. Assumptions for ANOVA were verified by visual inspection of normality and Levene’s test confirmed homogeneity of variances for all dependent variables (all *p*>0.05). Associations were explored using correlation and regression analyses. Multivariate logistic regression models were adjusted for age, sex, and disease group. A two-sided *p* value ≤0.05 was considered statistically significant. Given multiple secondary analyses, findings should be interpreted with caution due to potential type I error inflation.

## Results

### Study population and clinical baseline characteristics

Between May 2022 and October 2024, 103 patients were enrolled, exceeding the planned total sample size (*n*=95). Of these, 42 had LOPD, 20 had inclusion body myositis (IBM), 21 had facioscapulohumeral muscular dystrophy (FSHD), and 20 had spinal muscular atrophy type 3 (SMA3) (Table S1 and Fig S1). Disease confirmation results are summarized in supplements Table S2. Nineteen patients were excluded during screening due to depressive symptoms (Beck Depression Index-Fast Screen >=4) or inability to perform functional assessments. Demographic characteristics differed significantly across groups, particularly for age, age at symptom onset, and disease duration (Table 1). Baseline clinical characteristics are summarized in Table 2. Overall, no significant differences were observed between the four disease groups regarding functional assessment, as measured by the 6MWT %predicted (*p* = 0.169), or fatigue levels (FSS, *p* = 0.149). Similarly, depressive symptoms (BDI) and physical activity levels (RPAct) did not differ significantly across groups (p > 0.05). A significant intergroup difference was identified for the MRC-Score %predicted (*p* = 0.029), with FSHD and SMA3 patients showing numerically lower median scores compared to the LOPD and IBM groups. However, subsequent post hoc pairwise comparisons with the Bonferroni correction did not reveal any significant differences among specific groups.

**Table 1:** Baseline participant demographics

	Total (*n*=103)	LOPD (*n*=42)	IBM (*n*=20)	FSHD (*n*=21)	SMA3 (*n*=20)	*p* value^a^
Female n (%)	49 (48)	23 (55)	6 (30)	13 (62)	7 (35)	–
Age	51 (20−85)	48 (22−85)	70 (51−82)	41 (22−67)	42 (20−66)	**<0.01**
Age at symptom onset	33 (0−72)	27 (0−57)	61 (44−72)	27 (10−46)	15 (0−45)	**<0.01**
Disease duration [years]	18.9 (2−57)	19.2 (2−38)	8.8 (2−33)	19.9 (6−55)	28.3 (11−57)	**<0.001**
BMI [kg/cm^2^]	24 (12−36)	23 (12−36)	25 (15−33)	25 (16−33)	24 (14−33)	0.601

Baseline demographics by disease group. Data are presented as median (IQR) for non-normally distributed variables and ^a^analyzed via Kruskal–Wallis test

**Table 2:** Baseline clinical characteristics

	LOPD (*n*=42)	IBM (*n*=20)	FSHD (*n*=21)	SMA3 (*n*=20)	*p* value
6MWT, [%predicted]^a^	69.55 ± 27.10	65.97 ± 23.37	61.89 ± 25.35	52.05 ± 27.02	0.169
MRC-Score, [%predicted]^b^	84.21 (71.84–91.84)	86.85 (74.99–92.10)	71.58 (58.95–85.26)	70.53 (65.26–84.21)	**0.029**^**c**^
FSS^a^	33.31 ± 12.95	37.05 ± 18.32	42.35 ± 13.96	37.10 ± 13.15	0.149
BDI^b^	8.50 (7.00–11.00)	9.00 (7.00–14.50)	9.00 (7.25–12.00)	8.00 (7.00–12.00)	0.650
RPAct^a^	21.23 ± 2.15	20.33 ±5.46	12.89 ± 2.67	22.2 ± 3.25	0.088

Clinical baseline characteristics by disease group. Data are presented as mean ± standard deviation (SD) for normally distributed variables and median (IQR) for non-normally distributed variables. Group comparisons were performed using one-way ANOVA or the Kruskal–Wallis test, as appropriate. ^a^Variables with normal distribution analyzed via ANOVA. ^b^Variables with non-normal distribution analyzed via Kruskal–Wallis test. ^c^Although the overall Kruskal–Wallis test for MRC Scale % predicted was significant, post hoc pairwise comparisons with Bonferroni correction showed no significant differences between individual groups

### Primary outcome

**Fig. 1:**
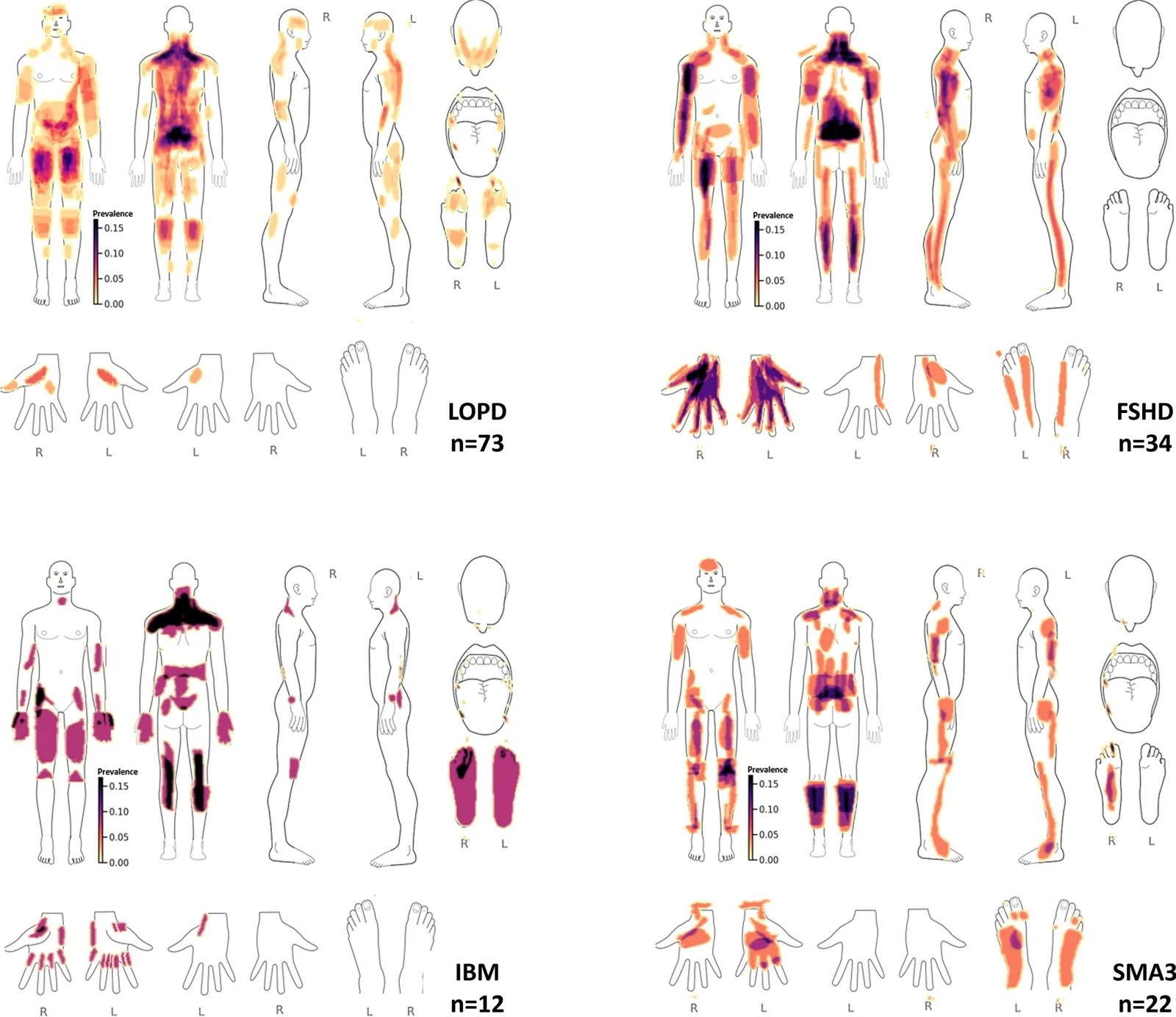
Heatmap of nociceptive pain drawings across the four disease groups. Heatmaps of pain distribution

Overall, 64.1% of all participants reported chronic nociceptive musculoskeletal pain. Within the LOPD group, 35 out of 42 patients (83.3%) were affected, with significantly higher prevalence compared to the other groups (*p*=0.005) and significantly more females reporting pain (Table 3 and supplements Table 3). Heatmaps of pain drawings illustrate the distribution of perceived musculoskeletal pain across disease groups. Patients were instructed to indicate the location of their pain on a body map, categorized by the following qualities: burning, pressing, pulling, or undefined (up to four pain qualities per patient). This resulted in a total of 144 pain drawings (LOPD *n*=76; FSHD *n*=34; SMA3 *n*=22; IBM *n*=12). Pain drawings were digitized and analyzed at a pixel-wise level using Pain2D-Software® to determine spatial prevalence (Fig. 1 and supplements Fig S2). Pain distribution patterns corresponded closely to disease-specific muscle involvement. In LOPD, pain was predominantly localized to proximal lower limbs and axial muscles. In contrast, pain on the day of the visit, captured by the BPI, was reported significantly lower than compared to the reported chronic pain. Subgroup analysis using the McNemar test revealed that this discrepancy was significant within the LOPD group (*p*<0.001) and the SMA3 group (*p*=0.016), whereas no significant differences between chronic and current pain were found for IBM (*p*=0.180) and FSHD (*p*=0.375). Due to BPI, pain intensity and interference scores were low across all groups on the day of the visit, with no significant differences between disease entities or sex (see Table S3). Pain characteristics and BPI scores are detailed in Table 3.

**Table 3:** Pain characteristics and BPI scores stratified by diagnostic group

	LOPD (*n*=42)	IBM (*n*=20)	FSHD (*n*=21)	SMA3 (*n*=20)	*p* value
Pain n (%) chronic pain	35 (83.3%)	8 (40%)	12 (57.1%)	11 (55%)	**0.005**^**1**^
BPI Pain today yes	13 (30.9%)	3 (15%)	9 (42.9%)	5 (25%)	0.255^2^
BPI pain intensity today	1.75 (1.32–3.07)	2.00 (0.60–3.89)	2.00 (1.34–3.11)	2.25 (1.89–2.70)	0.526^3^
BPI pain interference today	1.86 (1.13–2.96)	3.00 (1.58–7.30)	2.86 (1.57–5.26)	5.29 (1.24–7.22)	0.242^3^

Values are presented as n (%) or median (IQR). *p* values derived from ^1^Chi-square tests (Pain n), ^2^Fisher´s exact test (BPI Pain n) due to small cell counts (≤ 5 in 50% of cells) and ^3^Kruskal–Wallis test (BPI scores). *BPI* Brief Pain Inventory

### Secondary outcomes

#### Pain management

Pain was primarily managed with non-opioid analgesics, particularly NSAIDs, while opioid use was rare. Non-pharmacological approaches, especially thermotherapy, were frequently reported (supplements Table S4 and S5).

#### Pain in association with functional and structural parameters and laboratory tests

**Fig. 2:**
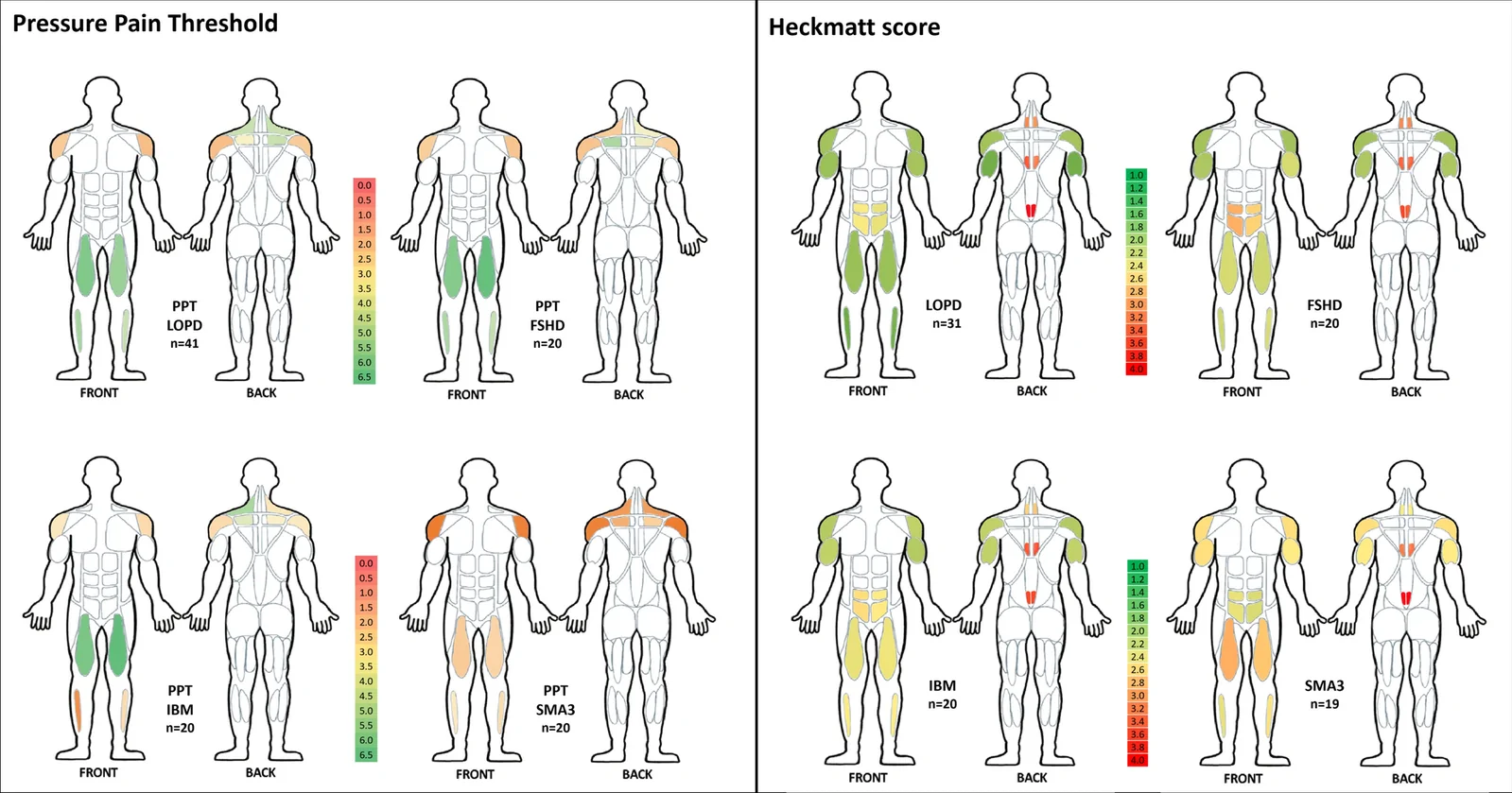
Heatmap of pressure pain threshold and Heckmatt score. Left: Anatomical heatmap of mean PPT values in kg/cm^2^. Right: mean Heckmatt scores across assessed muscles. Mean values are used for visualization purposes to illustrate trends across the ordinal 1–4 Heckmatt scale. Values for left and right figures are displayed in supplements Tables S6 and S10

Pressure pain thresholds differed across disease groups, with LOPD patients showing consistently higher thresholds than other groups, indicating reduced sensitivity to mechanical stimuli (Fig. 2 and supplemental Table S6 and Figure S3). However, these differences were not statistically robust after correction for multiple testing. No significant association was observed between the presence of pain and muscle strength (MRC), functional capacity (6MWT), or structural muscle alterations assessed by ultrasound. Similarly, biochemical parameters, including vitamin D and creatine kinase levels, were not associated with pain (supplements Table S7, S8, S9, 10 and Fig S4).

#### Genetic analyses

Genotype distributions for both ACTN3 and ACE polymorphisms were comparable across all four disease groups, with no statistically significant differences observed (*p*=0.653 and *p*=0.485, respectively; Table 4). In all groups, the heterozygous variants (RX and ID) represented the most frequent genotypes.

**Table 4:** ACTN3- and ACE-polymorphisms per disease group

Genotype	LOPD (*n*=42)	SMA3 (*n*=19)	IBM (*n*=20)	FSHD (*n*=20)	Total (*n*=101)	*p* value^1^
*ACTN3*						0.653
RX (het)	30 (71.4%)	15 (78.9%)	10 (50.0%)	10 (50.0%)	65 (64.4%)	
XX (hom)	5 (11.9%)	0 (0.0%)	3 (15.0%)	8 (40.0%)	16 (15.8%)	
RR (wt)	7 (16.7%)	4 (21.1%)	7 (35.0%)	2 (10.0%)	20 (19.8%)	
*ACE*						0.485
ID (het)	27 (64.3%)	9 (45.0%)	13 (65.0%)	9 (42.9%)	58 (56.3%)	
DD (hom)	5 (11.9%)	3 (15.0%)	2 (10.0%)	9 (42.9%)	19 (18.4%)	
II (wt)	10 (23.8%)	8 (40.0%)	5 (25.0%)	3 (14.3%)	26 (25.2%)	

Data are presented as absolute numbers and column percentages n (%). ^1^Fisher’s Exact test was used due small expected cell counts (< 5 in 50% of cells). *ACTN3* alpha-actinin-3 gene; *ACE* angiotensin-converting enzyme gene; *het* heterogenous; *hom* homozygous; *wt* wild type

### Correlation and association analyses

Statistical analysis revealed no significant association between the ACE or ACTN3 genotype and the reported chronic nociceptive pain (*p*=0.653) (Table 5).

**Table 5:** Association between ACTN3-/ACE genotype and the presence of chronic pain

Genotype	Pain + *n* (%)	Pain - *n* (%)	Total	*p* value	Adjusted residual
ACTN3				*p*=0.653^1^	
Homozygous (XX)	12 (75.0%)	4 (25.0%)	16		0.9
Heterozygous (RX)	42 (64.6%)	23 (35.4%)	65		−0.2
Wild-type (RR)	12 (60.0%)	8 (40.0%)	20		−0.6
ACE				*p*=0.485^1^	
Homozygous (DD)	13 (68.4%)	6 (31.6%)	19		0.3
Heterozygous (ID)	35 (60.3%)	23 (39.7%)	58		−1.2
Wild-type (II)	18 (75%)	6 (25.0%)	24		1.1

Data are presented as absolute numbers (n (%)). ^1^ Fisher’s exact test. Adjusted residuals > 1.96 indicate cells contributing significantly to the overall association. *ACTN3* alpha-actinin-3 gene; *ACE* angiotensin-converting enzyme gene; *het* heterogeneous; *hom* homozygous; *wt* wild type.

Further association analyses revealed no significant relationship between the presence or intensity of pain and any biochemical, demographic, or functional parameters, including vitamin D levels, disease duration, age, BMI, and motor function scores (6MWT, MRC) (supplements Tables S11 and S12). Specifically, within the LOPD group, no correlation was found between vitamin D levels and pain severity (*r*_*s*_=0.03*, p*=0.892). Consequently, as no trends were observed in the total cohort or in Vitamin D levels, no further subgroup-specific analyses were performed for the LOPD group. In multivariate logistic regression analysis, higher global PPT were independently associated with the presence of musculoskeletal pain (odds ratio 1.15, 95% CI 1.00–1.32; *p*=0.046), after adjustment for disease group, age, and sex.

The ACE and ACTN3 polymorphisms showed no significant association with the age of symptom onset, muscle strength (MRC), or functional capacity (6MWT) in either the total cohort or the LOPD subgroup. Although ACE genotypes were significantly associated with differences in creatine kinase (CK) levels (*p*=0.014), with the II-genotype exhibiting higher values, this biochemical variation did not translate into detectable differences in clinical motor performance (supplements Table S12).

A significant moderate-to-strong negative correlation was found between 6MWT performance and a lower-limb composite Heckmatt score (Spearman´s *rs*=− 0.574; *p*<0.001), indicating that an increased echogenicity and structural degeneration of the lower extremities are strongly linked to reduced submaximal functional capacity (supplements Fig S5). In multivariate logistic regression analyses adjusted for age, sex, and disease group, neither 6MWT % predicted, MRC sum score nor global mean Heckmatt score was associated with musculoskeletal pain (see Table S8). Regarding patient-reported outcomes, no significant difference in FSS score was observed between patients with and without nociceptive pain (*p* = 0.116). However, fatigue severity showed a weak positive correlation with pain intensity (Spearman’s rₛ = 0.220, *p* = 0.026) (see Table S11).

## Discussion

This is the first multicentre cross-sectional study to systematically evaluate nociceptive pain in patients with late-onset Pompe disease (LOPD) by integrating patient-reported outcomes with clinical assessments of muscle function, structure, and pain perception, alongside laboratory modifiers. To better contextualize the pain profile of LOPD, these findings were compared with other neuromuscular disorders sharing a phenotype of slowly progressive, predominantly proximal and axial muscle weakness, but differing in their underlying pathology.

Musculoskeletal pain was highly prevalent in LOPD and occurred significantly more frequently than in comparable neuromuscular diseases. However, pain was not associated with objective measures of muscle strength, structural muscle alterations assessed by ultrasound, or biochemical parameters.

Our observed prevalence of nociceptive pain in LOPD is consistent with the previous reports highlighting pain as a relevant but underrecognized symptom in Pompe disease [12, 20, 21, 30, 35]. The spatial distribution of pain, predominantly affecting the proximal lower limbs and axial musculature, reflects the typical pattern of muscle involvement in LOPD. A key finding of this study is the absence of a significant relationship between pain and objective markers of disease severity, including muscle strength (MRC), functional capacity (6MWT), and muscle ultrasound findings. Notably, the lack of association between perceived pain and Heckmatt scores argues against a direct link between structural muscle degeneration and nociceptive pain generation. Similarly, biochemical parameters, including creatine kinase and vitamin D levels, were not associated with pain. These findings suggest that commonly assumed contributors to musculoskeletal pain, such as muscle damage or metabolic alterations, do not sufficiently explain pain perception in LOPD.

LOPD patients exhibited comparatively higher pressure pain thresholds across multiple muscle groups, indicating reduced sensitivity to mechanical stimuli. This may reflect altered central pain processing in the context of chronic, slowly progressive neuromuscular disease. In contrast to preliminary observations, we found no association between ACTN3 or ACE polymorphisms and chronic nociceptive musculoskeletal pain. While ACTN3 XX may influence muscle resilience, our data suggest that in diseases like LOPD, the primary pathology and mechanical factors overshadow these genetic modifiers [10]. Although ACE genotype was associated with creatine kinase levels, this did not translate into differences in motor performance, and its clinical relevance remains uncertain.

The inclusion of disease control groups (IBM, FSHD, SMA3) provides important context for interpreting these findings in LOPD. Despite different pathophysiological mechanisms, pain quality and severity were broadly comparable across groups. The lack of a clear association between pain and disease duration or functional impairment across all groups suggests that chronic neuromuscular disease per se does not inevitably lead to increased pain burden. These findings further support the concept that pain in neuromuscular disorders is multifactorial and not solely determined by the degree of muscle pathology but may arise from predominantly regional muscle and musculoskeletal imbalances rather than structural muscle tissue alteration. Although muscle biopsy was not performed in this study, future studies incorporating histopathological analyses, including muscle fiber distribution, may help clarify whether specific structural muscle alterations contribute to pain in LOPD.

A key finding in our study is the discrepancy between the high prevalence of reported chronic musculoskeletal pain and the significantly lower frequency of pain on the day of assessment in LOPD and SMA3. This indicates that single-point assessments such as the BPI may underestimate the true pain burden. Pain in these patients appears to be fluctuating and activity-dependent, which may not be captured during single clinical visits. This temporal dissociation may also explain the lack of correlation between pain intensity and objective functional measures. Consequently, from a clinical perspective, the dissociation between pain and objective disease severity highlights the need for independent assessment and management of pain in LOPD, as well as repeated clinical reassessment over time. Standard neuromuscular evaluations focusing on strength and function do not adequately reflect the patient’s pain burden, supporting the routine use of dedicated pain assessment tools.

Fatigue may represent a relevant factor influencing pain perception in patients with neuromuscular disorders. Although fatigue severity was not associated with the presence of nociceptive pain, higher FSS scores were weakly associated with greater pain intensity, suggesting that fatigue may contribute to the subjective burden of pain rather than its occurrence.

This study has several limitations. The sample size, although exceeding initial targets, remains limited for subgroup analyses. The cross-sectional design precludes conclusions on causality or longitudinal progression. While adjustments for multiple testing were applied in primary analyses, the exploratory nature of secondary outcomes increases the risk of type I error. Finally, pain remains a subjective construct, and psychosocial factors beyond depression were not systematically assessed.

In conclusion, musculoskeletal pain is a frequent but largely independent clinical feature in LOPD that is not explained by muscle strength, structural damage, biochemical parameters, or common genetic modifiers. These findings support the concept of pain as a distinct clinical domain requiring dedicated assessment and management.

## Supplementary Information

Below is the link to the electronic supplementary material.

Supplementary file1 (PDF 670 KB)

## Data Availability

The anonymized participant data presented here are available upon request from the correspondent author (stephan.wenninger@med.uni-muenchen.de).

## Declarations

### Study registration

This study was registered at clinicaltrials.gov, number NCT05272969.
