# Supplementary material for: Nociceptive pain in late-onset Pompe disease: prevalence, distribution, and clinical correlates
Source: J Neurol. 2026 Aug 25;273(9):544. doi: 10.1007/s00415-026-14071-x (PMC13506554; doi:10.1007/s00415-026-14071-x)
Supplement: Supplementary file 1 [file 415_2026_14071_MOESM1_ESM.pdf]

Nociceptive Pain in Late-Onset Pompe Disease - Prevalence, Pattern, and Clinical Correlates

Stephan Wenninger*[1], Marcela Arndt*[1], Daniel H Mendelsohn[1], Corinna Wirner-Piotrowski[1], Marko Mijic[1], Natalia Garcia-Angarita[1], Kristina Gutschmidt[1], Christoph Schmidt[2], Lisa Winkler[2], Dieter Gläser[2], Karl Christian Knop[3], Matthias Spranger[4], Frank Klawonn[5], Lea Eileen Brauner[5], and Benedikt Schoser[1]

Manuscript Supplements

Table S1: Recruitment status

Disease	Number anticipated	Number enrolled	% of anticipated
**LOPD**	50	42	84
**IBM**	15	20	133
**FSHD**	15	21	140
**SMA3**	15	20	133
**Total**	95	103	108

Abbreviations: LOPD – Late onset Pompe Disease; IBM – Inclusion Body Myositis; FSHD – Facioscapulohumeral muscular dystrophy; SMA3 – Spinal muscular atrophy Type 3.

Table S2: Disease confirmation

Disease	Confirming diagnostic tests	n	%
LOPD	Genotype		
	• c.-32-13T>G/ c.525del	8	19.0
	• c.-32-13T>G/ c.510C>T	3	7.1
	• c.-32-13T>G/ c.1438-1G>C	2	4.8
	• c.-32-13T>G/ c.118C>T	2	4.8
	• c.-32-13T>G/ c.(2646+1_*2747-1)*del	2	4.8
	• c.-32-13T>G/ c.1548G>A	2	4.8
	• c.-32-13T>G/ c.2482_2464del	2	4.8
	• c.-32-13T>G/ c.925G>A	1	2.4
	• c.-32-13T>G/ c.1441T>C	1	2.4
	• c.-32-13T>G/ c.1818A>G	1	2.4
	• c.-32-13T>G/ c.546+4T>G	1	2.4
	• c.-32-13T>G/ c.743T>C	1	2.4
	• c.-32-13T>G/ c.2501_2502del	1	2.4
	• c.-32-13T>G/ c.2281delinsAT	1	2.4
	• c.-32-13T>G/ c.1721T>C	1	2.4
	• c.719C>T/ c.877G>A	1	2.4
	• c.1933G>A/ c.2702T>A	1	2.4
	• c.-32-13T>G/ c.877G>A	1	2.4
	• c.-32-13T>G/ c.1456G>C	1	2.4
	• c.-32-13T>G/ Del. Exon 18	1	2.4
	• c.-32-13T>G/ c.2746G>T	1	2.4
	• c.-32-13T>G/ c.2241_2242insCCTC	1	2.4
	• c.1832C>T/ c.1912G>T	1	2.4
	• c.-32-13T>G/ c.2242delinsCCTCC	1	2.4
	• c.-32-13T>G/ c.1927G>A	1	2.4
	• c.-32-13T>G/ Del. Exon 20	1	2.4
	• c.-32-13T>G/ c.1643T>C	1	2.4
	Muscle biopsy		
	vacuolar myopathy	8	19.0
IBM	• Muscle biopsy	20	100
	• Clinical phenotype	20	100
FSHD	Genotype		
	FSHD1,4qA-Haplotype, D4Z4-Repeat-Contraction (≤10 RU)	20	95.2
	• 1–3 Repeats	3	15.0
	• 4-6 Repeats	9	45.0
	• 7-10 Repeats	8	40.0
	FSHD2: „SMCHD1 splice-site variant (c.1463+5G>A) with D4Z4 hypomethylation (27%), consistent with FSHD2	1	4.8
SMA3	Genotype		
	Deletion in Exon 7 and 8 in SMN1 gene	20	100
	• 3 SMN2 copies	8	40
	• 4 SM2 copies	12	60

Genetic analysis for LOPD revealed that 88.1% (38/42) of the patients carried the common leaky splice-site mutation c.-32-13T>G (IVS1-13T>G) in a compound-heterozygous state. The most frequent second pathogenic variant was c.525del (found in 21.1% of c.-32-13T>G carriers), followed by c.510C>T and c.1438-1G>C, reflecting a typical Late-Onset Pompe Disease (LOPD) genotype distribution. Genetic analysis for FSHD: D4Z4 repeat size was expressed as repeat units (RU). For Southern blot results, RU were estimated from EcoRI fragment size using an approximate conversion (1 RU ≈ 3.3 kb after subtraction of ~5 kb flanking sequence).

Table S3: Prevalence of pain across disease groups and gender

Disease	Pain female yes n (%)	Pain male yes n (%)	p-value*
**LOPD (n=35)**	22 (62.9%)	13 (37.1%)	0.034
**IBM (n=8)**	3 (37.5%)	5 (62,5%)	0.642
**FSHD (n=12)**	9 (75.0%)	3 (25.0%)	0.365
**SMA3 (n=11)**	5 (45.5%)	6 (54.5%)	0.633

*Fishers exact test due to small cell counts ≤5 in 50% of cells

Table S4: Analgesic medication across disease groups

Treatment[1]	LOPD n=42	IBM n=20	FSHD n=21	SMA3 n=20	Total n=103
**Ibuprofen**	8 (19.0%)	3 (15.0%)	2 (9.5%)	4 (20.0%)	17 (16.5%)
**Paracetamol**	4 (9.5%)	2 (10.0%)	3 (14.3%)	3 (15.0%)	12 (11.7%)
**Metamizol**	5 (11.9%)	2 (10.0%)	2 (9.5%)	3 (15.0%)	12 (11.7%)
**Diclofenac**	3 (7.1%)	1 (5.0%)	--	1 (5.0%)	5 (4.9%)
**Duloxetin[2]**	1 (2.4%)	--	1 (4.8%)	1 (5.0%)	3 (2.9%)
**Morphine**	--	--	--	1 (5.0%)	1 (1.0%)
**Tramadol**	--	--	--	1 (5.0%)	1 (1.0%)
**Tilidine**	--	--	--	1 (5.0%)	1 (1.0%)
**THC/CBD**	--	--	1 (4.8%)	1 (5.0%)	2 (1.9%)
**Pregabalin[2]**	1 (2.4%)	--	1 (4.8%)	--	2 (1.9%)
**Amitryptiline[2]**	1 (2.4%)	--	--	--	1 (1.0%)
**Venlafaxin[2]**	1 (2.4%)	--	--	--	1 (1.0%)
**Oxycodone**	--	--	1 (4.8%)	--	1 (1.0%)

1: multiple selections allowed. 2: medication used as an analgesic

Table S5: other pain treatment

Treatment[1]	LOPD n=42	IBM n=20	FSHD n=21	SMA3 n=20	Total n=103
**Accupuncture**	2 (11.1%)	1 (50.0%)	1 (16.7%)	1 (33.3%)	5 (17.2%)
**Psychotherapy**	2 (11.1%)	--	--	--	2 (6.9%)
**Cold/Warmth therapy**	13 (72.2%)	1 (50.0%)	4 (66.7%)	2 (66.7%)	20 (69.0%)
**Electro therapy**	1 (5.6%)	--	--	--	1 (3.4%)
**Magentic resonance therapy**	--	--	1 (16.7%)	--	1 (3.4%)
**Total**	18	2	6	3	29

1: multiple selections allowed

Table S6: Pressure Pain Threshold (PPT) measurement

	LOPD n=41	IBM n=20	FSHD n=20	SMA3 n=20	p-value
**Trazpezius left**	5.4 (2.90 - 7.50)	5.5 (3.35 - 7.10)	4.4 (2.55 - 6.25)	3.6 (3.28 - 6.68)	0.752
**Trapezius right**	5.4 (3.20 - 7.50)	4.8 (3.12 - 6.83)	5.1 (2.98 - 6.58)	3.7 (2.89 - 5.75)	0.346
**Supraspinatus left**	5.0 (3.33 - 8.68)	5.2 (3.60 - 6.97)	5.7 (2.58 - 7.85)	4.0 (3.18 - 5.70)	0.502
**Supraspinatus right**	5.5 (3.13 - 9.53)	5.0 (3.55 - 7.65)	5.1 (2.75 - 7.15)	4.4 (3.38 - 6.05)	0.840
**Deltoid left**	4.2 (2.90 - 7.23)	4.6 (3.15 - 5.80)	4.3 (2.88 - 6.30)	3.3 (2.50 - 4.93)	0.425
**Deltoid right**	4.4 (2.60 - 6.28)	4.8 (3.42 - 6.52)	4.4 (2.48 - 5.50)	3.2 (2.58 - 5.08)	0.358
**Rectus fem left**	5.8 (4.23 - 10.98)	6.3 (4.57 - 9.50)	6.2 (3.90 - 9.10)	4.5 (3.4 - 6.025)	0.124
**Rectus fem right**	6.0 (4.30 - 11.65)	6.1 (3.95 - 8.75)	6.0 (4.38 - 7.88)	4.4 (3.35 - 5.95)	0.125
**Tibialis ant. left**	5.4 (3.93 – 10.00)	4.6 (3.35 - 7.40)	5.5 (3.53 - 8.35)	4.9 (3.38 - 5.95)	0.240
**Tibialis ant. right**	5.7 (4.53 - 10.75)	3.5 (2.85 - 7.83)	5.7 (3.75 - 7.63)	4.8 (3.70 - 5.58)	0.033*

All values in units of force, kg/cm² and median (IQR). Higher PPT values indicate a greater tolerance to pressure pain (i.e., less sensitivity), while lower values suggest increased pain sensitivity. P-values were calculated using the Kruskal-Wallis test for independent samples, with $p<0.05$ considered statistically significant. *After applying the conservative Bonferroni correction for multiple comparisons, no specific pairwise difference reached statistical significance.

Table S7: Functional assessments per disease group

	LOPD n=42	IBM n=20	FSHD n=21	SMA3 n=20	P value
6MWT [m][1]	430.5 ± 166.3	337.8 ± 122.6	391.2 ± 173.9	352.1 ± 184.7	0.212
6MWT [%pred][1]	68.4 ± 25.4	66.0 ± 23.4	61.9 ± 25.4	52.1 ± 27.0	0.166
MRC score[2]	59.50 (71.00-85.00)	81.50 (75.00-86.00)	65.50 (56.00-81.00)	67.00 (64.00-80.00)	0.044[3]
MRC [%pred][2]	84.21 (76.84-90.53)	86.85 (78.95-90.53)	71.58 (64.21-85.26)	70.53 (57.37-84.21)	0.029[3]

Data are presented as mean ± SD for normally distributed variables and median (Interquartile range, IQR) for non-normally distributed variables. [1]Group comparisons were performed using one-way ANOVA for normally distributed variables (e.g., 6MWT) and the [2]Kruskal-Wallis test for non-normally distributed variables (e.g., MRC scores). Post-hoc pairwise comparisons were conducted using Dunn's test with Bonferroni correction. [3]While the Kruskal-Wallis test indicated an overall difference in MRC score and MRC %predicted across groups, individual pairwise comparisons did not reach statistical significance after Bonferroni correction. Abbreviations: MRC – Medical Research Council; 6MWT – 6-Minute Walk Test; %pred. - % predicted.

Table S8: Association of musculoskeletal pain with functional assessments

	Pain-	Pain+	Mann-Whitney U	p-value	Adjusted OR (95% CI)	Adjusted p-value
6MWT %pred, median (Q1–Q3)	66.45 (44.75–82.81)	62.65 (34.20–85.86)	741.0	0.992	1.000 (0.096–1.003)	0.833
MRC% pred, median (Q1–Q3)	79.0 (67.0–85.0)	77.0 (67.0–88.0)	1007.5	0.405	0.983 (0.951–1.017)	0.320
Global Heckmatt score, median (Q1–Q3)	2.72 (2.56–3.28)	2.72 (2.11–3.17)	996.0	0.580	1.284 (0.812–5.872)	0.122

Data are presented as median (Interquartile range, IQR) for non-normally distributed variables. Group comparisons were performed using the Kruskal-Wallis test, with $p<0.05$ considered statistically significant. Abbreviations: MRC – Medical Research Council; 6MWT – 6-Minute Walk Test; %pred. - % predicted; OR - odds ratio; CI – confidence interval.

Table S9: Laboratory analyses

	**LOPD n=32**	**IBM n=20**	**FSHD n=20**	**SMA3 n=20**	**p-value**
Vit. D [ng/ml]	24.55 (16.25-32.70)	30.70 (24.20-43.50)	24.80 (22.30-31.80)	28.50 (20.95-42.5)	0.532
Mg^{2+} [mmol/l]	0.82 (0.79-0.85)	0.82 (0.79-0.88)	0.85 (0.81-0.89)	0.86 (0.79-0.91)	0.344
P^{+} [mg/dl]	3.5 ± 0.58	3.2 ± 0.50	3.3 ± 0.53	3.6 ± 0.52	0.129
Creatine Kinase [U/l]	332.00 (216-525)	243.00 (168-408)	270.00 (190-398)	418.00 (252-665)	0.092

Data are presented as mean ± standard deviation (SD) for normally distributed variables and median (IQR) for non-normally distributed variables. Group comparisons were performed using one-way ANOVA or Kruskal-Wallis test for continuous variables. Abbreviations: Vit. D – Vitamin D; Mg^{2+} - Magnesium; P+ - Phosphate; CK – Creatine Kinase.

Table S10: Muscle ultrasound assessments

	LOPD n=31	IBM n=20	FSHD n=20	SMA3 n=19	p-value
Delt_R	2.2 ± 1.31	2.3 ± 0.66	2.2 ± 1.10	2.9 ± 1.02	0.033[#]
Delt_L	2.2 ± 1.27	2.4 ± 0.75	2.2 ± 1.02	2.9 ± 1.02	0.026[#]
TricBr_R	1.8 ± 1.06	2.4 ± 0.94	2.3 ± 1.01	2.8 ± 0.92	<0.001[#]
TricBr_L	1.8 ± 1.08	2.5 ± 0.83	2.3 ± 1.08	2.8 ± 0.92	<0.001[#]
BBr_R	2.1 ± 1.29	2.4 ± 0.99	2.5 ± 1.28	2.9 ± 1.02	0.079
BBr_L	2.2 ± 1.30	2.3 ± 0.83	2.6 ± 1.40	2.8 ± 1.03	0.164
RAbd_R	2.8 ± 1.42	2.9 ± 0.55	3.1 ± 0.86	2.7 ± 0.94	0.290
RAbd_L	2.7 ± 1.39	2.9 ± 0.49	3.0 ± 0.93	2.6 ± 0.89	0.384
RF_R	2.1 ± 1.24	2.7 ± 0.89	2.6 ± 1.08	3.1 ± 1.00	0.003[#]
RF_L	2.1 ± 1.22	2.7 ± 0.88	2.6 ± 1.08	3.0 ± 0.93	0.004[#]
TA_R	1.8 ± 1.24	2.9 ± 1.04	2.5 ± 1.16	2.7 ± 1.36	0.010*
TA_L	1.8 ± 1.19	2.8 ± 1.01	2.6 ± 1.29	2.9 ± 1.37	0.003[#]*
C6_R	3.2 ± 1.70	3.0 ± 1.70	3.3 ± 1.24	2.8 ± 1.22	0.416
C6_L	3.3 ± 1.73	3.0 ± 1.73	3.3 ± 1.24	2.8 ± 1.22	0.217
TH6_R	3.3 ± 1.70	3.5 ± 1.70	3.4 ± 1.34	3.4 ± 1.28	0.905
TH6_L	3.4 ± 1.71	3.5 ± 1.71	3.4 ± 1.34	3.3 ± 1.27	0.989
L5_R	3.8 ± 1.80	3.6 ± 1.80	3.5 ± 1.15	3.8 ± 1.09	0.034**
L5_L	3.8 ± 1.79	3.6 ± 1.79	3.6 ± 1.12	3.8 ± 1.09	0.079

1.00
1.20
1.40
1.60
1.80
2.00
2.20
2.40
2.60
2.80
3.00
3.20
3.40
3.60
3.80
4.00

Muscle ultrasound assessments across disease groups. Data are presented as mean ± standard deviation (SD) for the Heckmatt scale (1–4) to illustrate distribution. P-values were calculated using the Kruskal-Wallis test to compare LOPD, IBM, FSHD, and SMA3 cohorts. p-values in the right column refer to the global Kruskal-Wallis test across all four groups. Post-hoc pairwise comparisons (Games-Howell) specifically comparing LOPD to the other cohorts revealed significant differences ($p < 0.05$) for: [#]LOPD vs. SMA3: Delt_R/L, TricBr_R/L, RF_R/L, TA_L and *LOPD vs. IBM: TA_R/L. **Not valid after Games-Howell-Correction. All reported post-hoc p-values are adjusted for multiple testing. Abbreviations: Delt – Deltoid muscle; TricBr – Triceps brachii muscle; BBr – Biceps brachii muscle; Rabd – Rectus abdominis muscle; RF – Rectus femoris muscle;

TA – Tibialis anterior muscle; C7 – Paraspinal cervical muscle C7; TH6 – Paraspinal thoracic muscle Th6; L5 – Paraspinal lumbar muscle L5; R – Right; L – Left.

Table S11: Association analyses between reported nociceptive pain and different variables.

Parameter	Pain (yes)[a]	Pain intensity (BPI)[b]	
	p-value	Correlation (r_s)	p-value
**Biochemical**			
Vitamin D [ng/ml]	0.997	0.07	0.522
**Demographic**			
Disease duration [years]	0.146	0.09	0.423
Age [years]	0.057[c]	-0.13	0.205
BMI [kg/m^2]	0.103[c]	-0.003	0.973
**Motor function**			
6MWT [%predicted]	0.788[c]	-0.01	0.940
MRC [%predicted]	0.405	-0.09	0.381
**Patient-reported outcome**			
Fatigue Severity Scale (FSS)	0.116[c]	0.220	0.026

[a] Analysis of differences between patients with and without pain (total cohort). [b] Correlation between BPI pain intensity and the respective variable (total cohort). [c] Determined by independent t-test (normally distributed); all other p-values in this column determined by Mann-Whitney U test. Spearman's rho (r_s) was used for all correlation analyses. Abbreviations: BMI – body mass index; 6MWT: 6-minute walk test; MRC: Medical Research Council sum score. BPI: Brief Pain Inventory. FSS: Fatigue Severity Scale.

Table S12: Association analyses ACTN3/ACE-Polymorphism

	ACTN3				ACE			
	XX (hom)	RX (het)	RR (wt)	p-value	ID (het)	DD (hom)	II (wt)	p-value
6MWT [%pred.]	53.40±27.02	64.95±26.62	67.48±24.44	0.661	64.45±28.91	56.18±24.35	66.96±22.14	0.609
MRC [%pred.]	68.16 (57.37-87.37)	78.95 (67.96-90.00)	87.37 (76.84-92.63)	0.117[a]	78.95 (67.37-90.53)	70.53 (64.21-85.26)	84.21 (71.58-87.37)	0.282[a]
CK	307.00 (118.00-498.00)	277.00 (188.00-461.00)	393.00 (190.00-559.00)	0.695[a]	270.50 (179-404)	283.50 (159.00-478.00)	438.00 (225.00-762.00)	0.014[a]
Vit. D [ng/ml]	23.20 (17.80-33.50)	28.50 (20.30-35.50)	28.10 (22.30-32.30)	0.414[a]	28.20 (21.30-32.40)	25.35 (19.40-35.10)	26.30 (19.70-38.70)	0.970[a]

Data are presented as mean ± SD for normally distributed variables and median (IQR) for non-normally distributed variables. Group comparisons were performed using one-way ANOVA or Kruskal-Wallis test for continuous variables. [a] Kruskal-Wallis test. Abbreviations: 6MWT: 6-minute walk test; MRC: Medical Research Council sum score; %pred. - % predicted; Vit. D – Vitamin D; CK – Creatine Kinase.

Figure S1: Recruitment flow

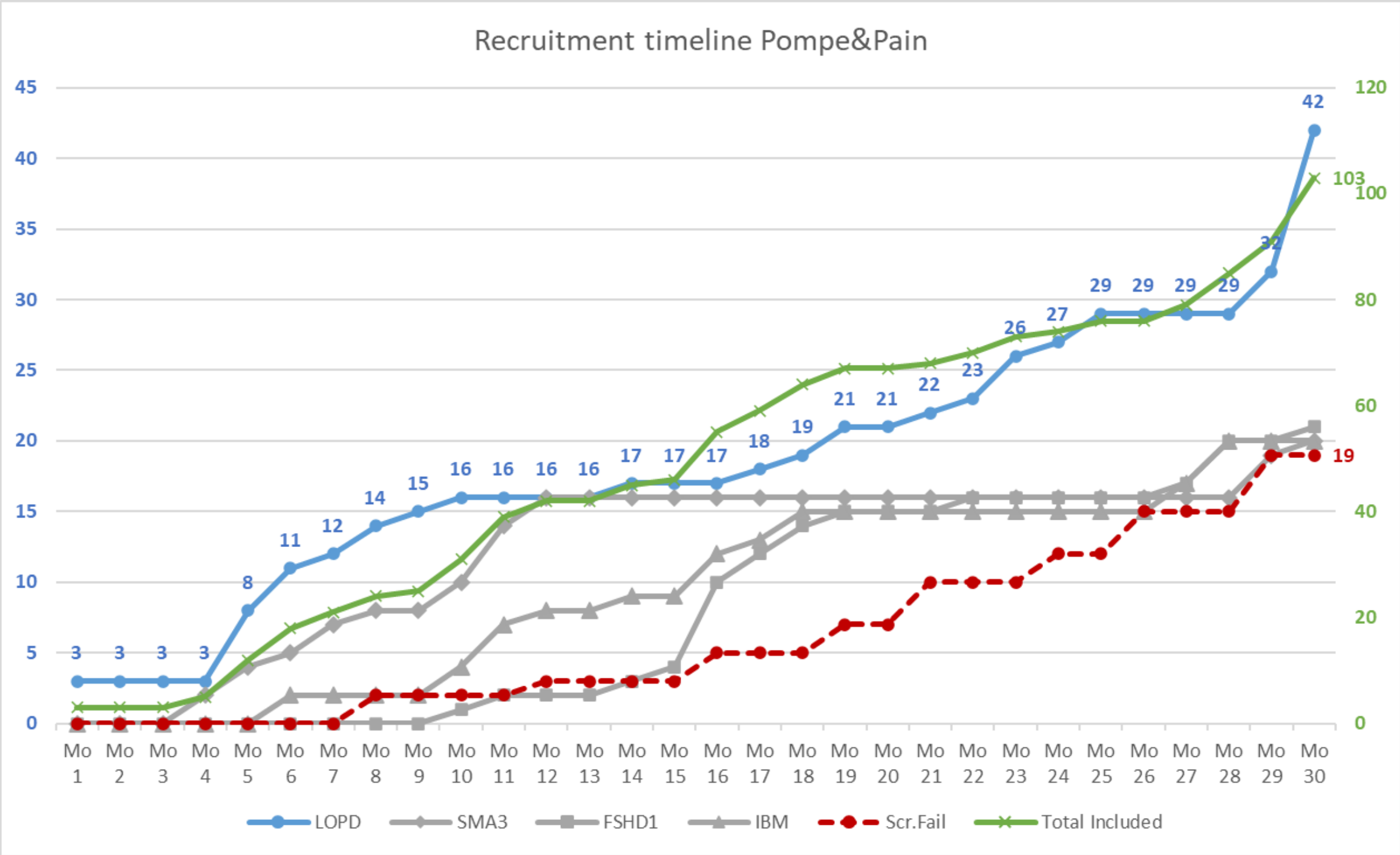

Due to protocol exclusion criteria, 19 Patients could not be included, 13 due to a high score in Beck Depression Index – Fast Screen (BDI-FS) of more ≥ 4 points at screening, 6 patients were not able to walk (complete wheelchair bound). Mo=Month; ScrFail = Screening Failure.

Figure S2: Pain quality per disease group

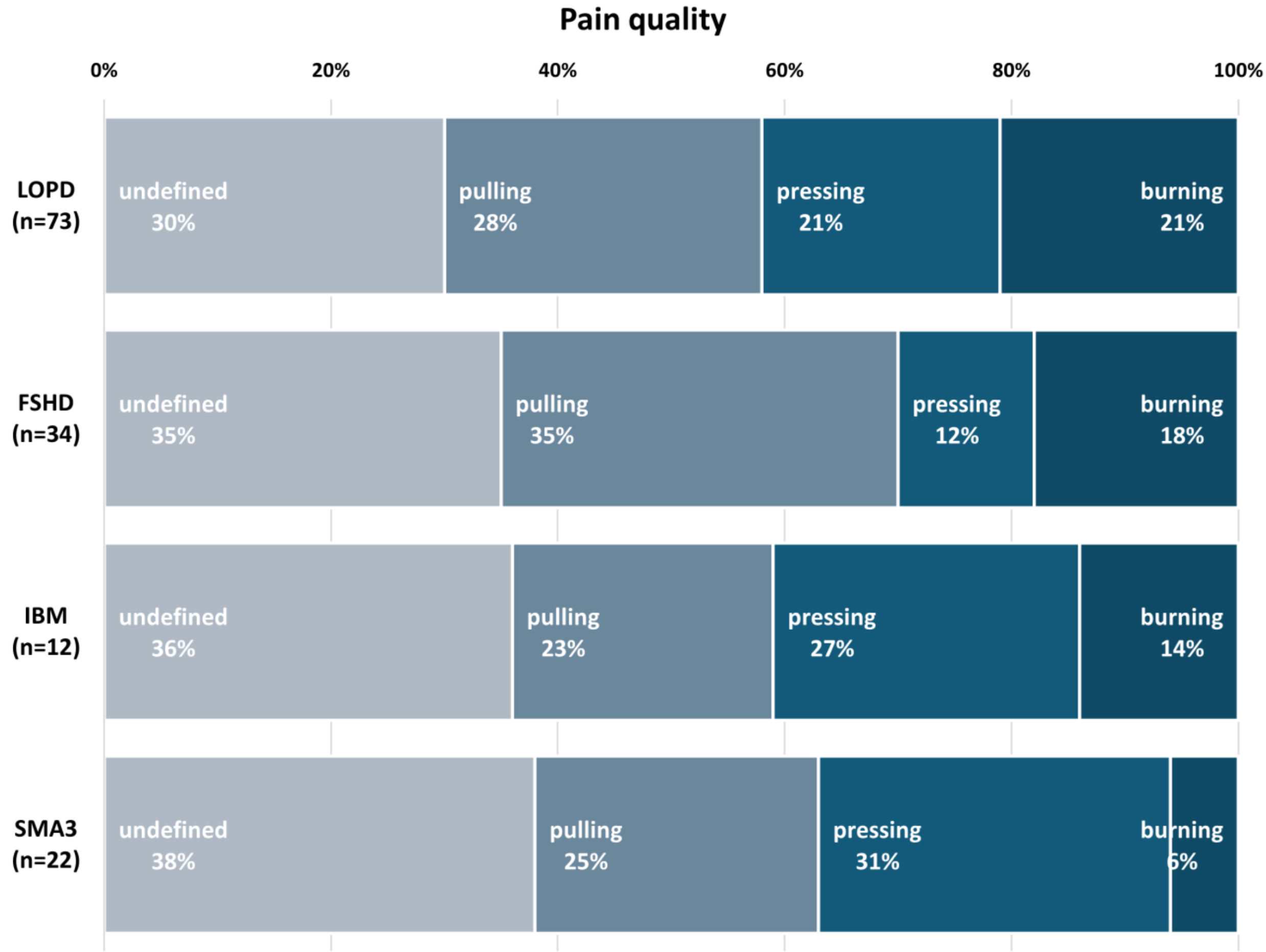

Stacked bar chart illustrating the relative frequency of different pain qualities within each disease cohort, with percentages based on the respective sample sizes (n). Fisher´s exact test revealed no significant differences in quality of pain between the disease groups ($p>0.05$).

Figure S3: Pressure Pain Threshold (PPT) across disease groups

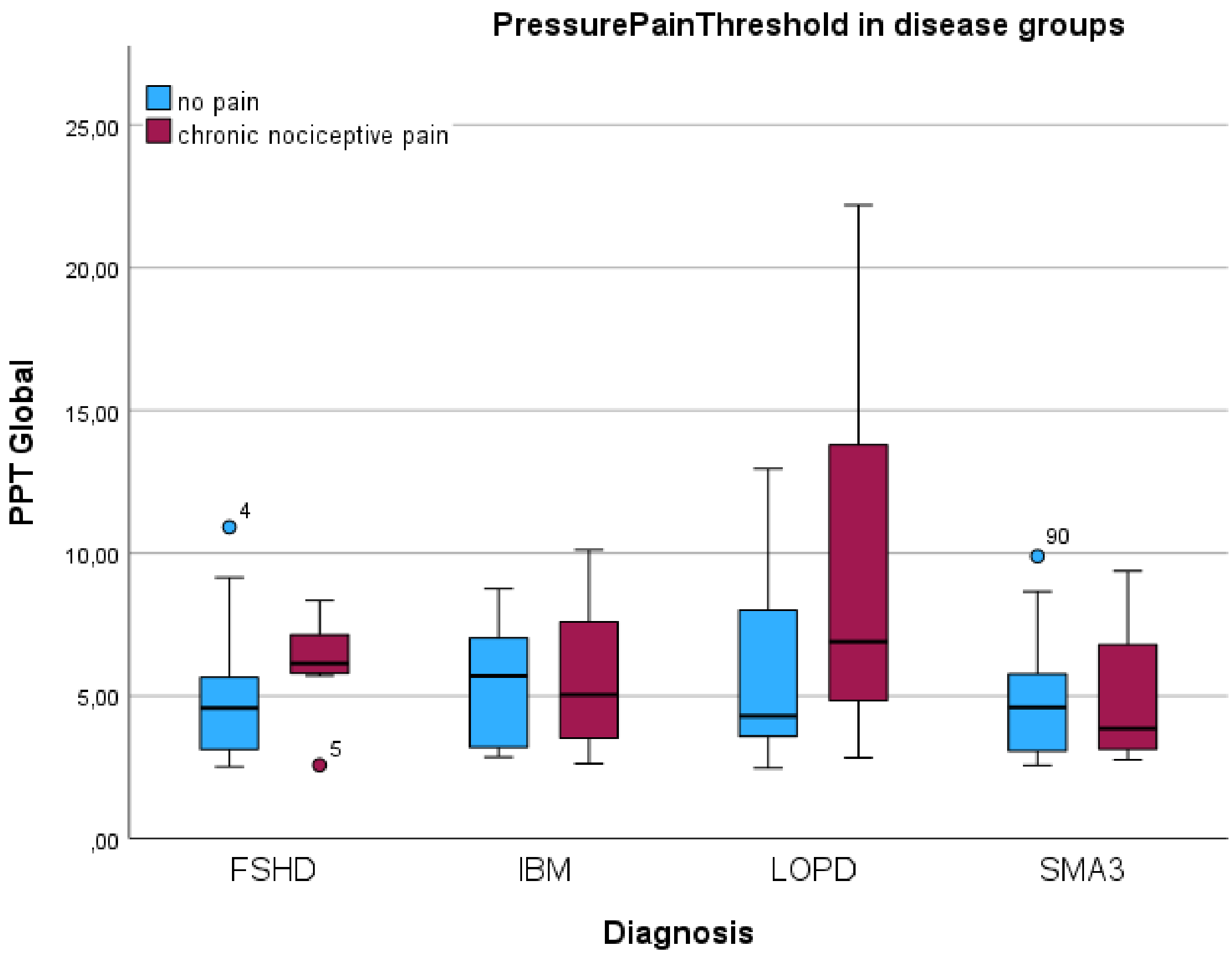

Figure S4: Laboratory analysis of Vitamin D, Magnesium, Phosphate and CK-Levels across disease groups.

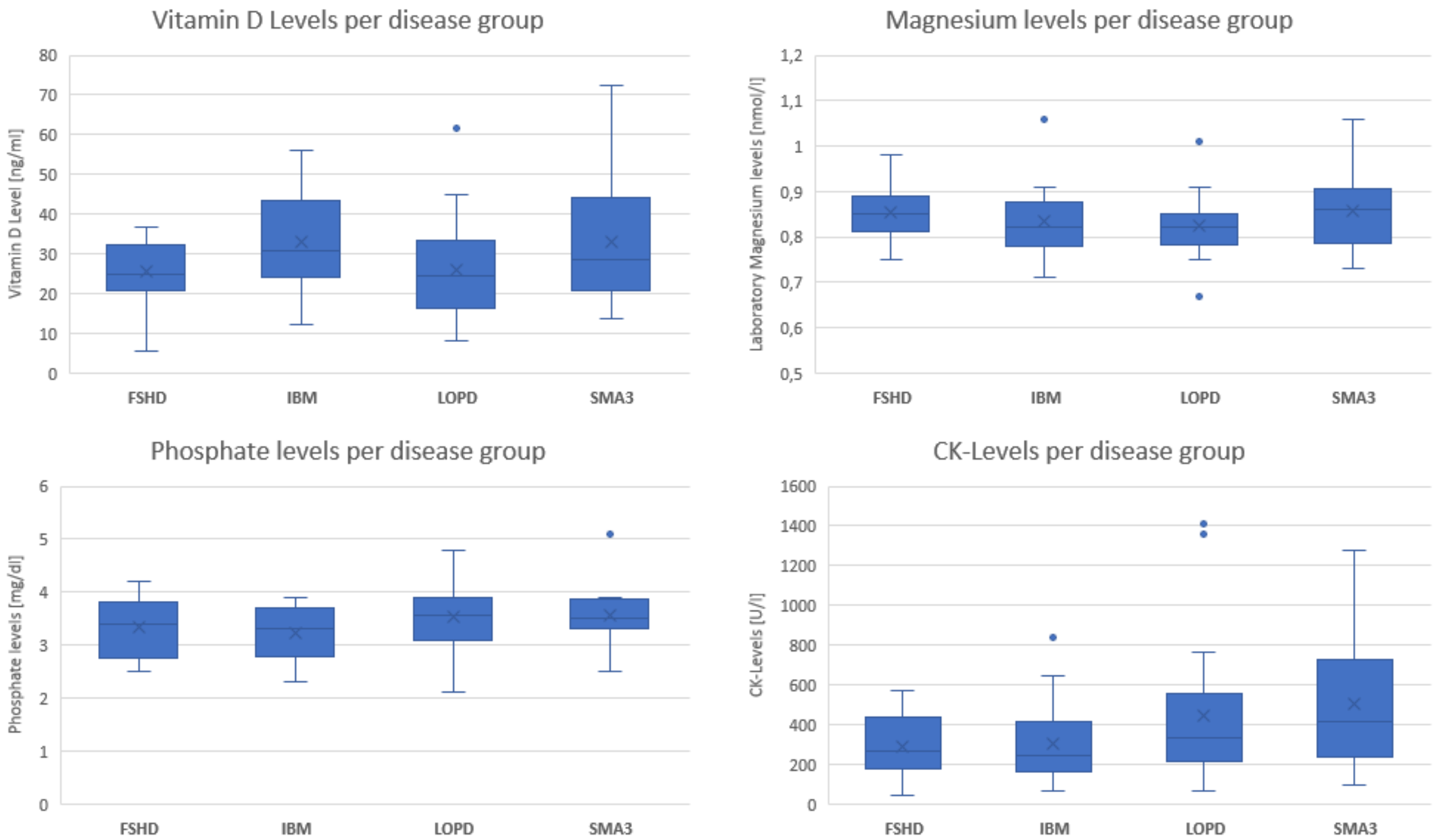

Figure S5: Correlation between lower limb muscle echogenicity and functional capacity.

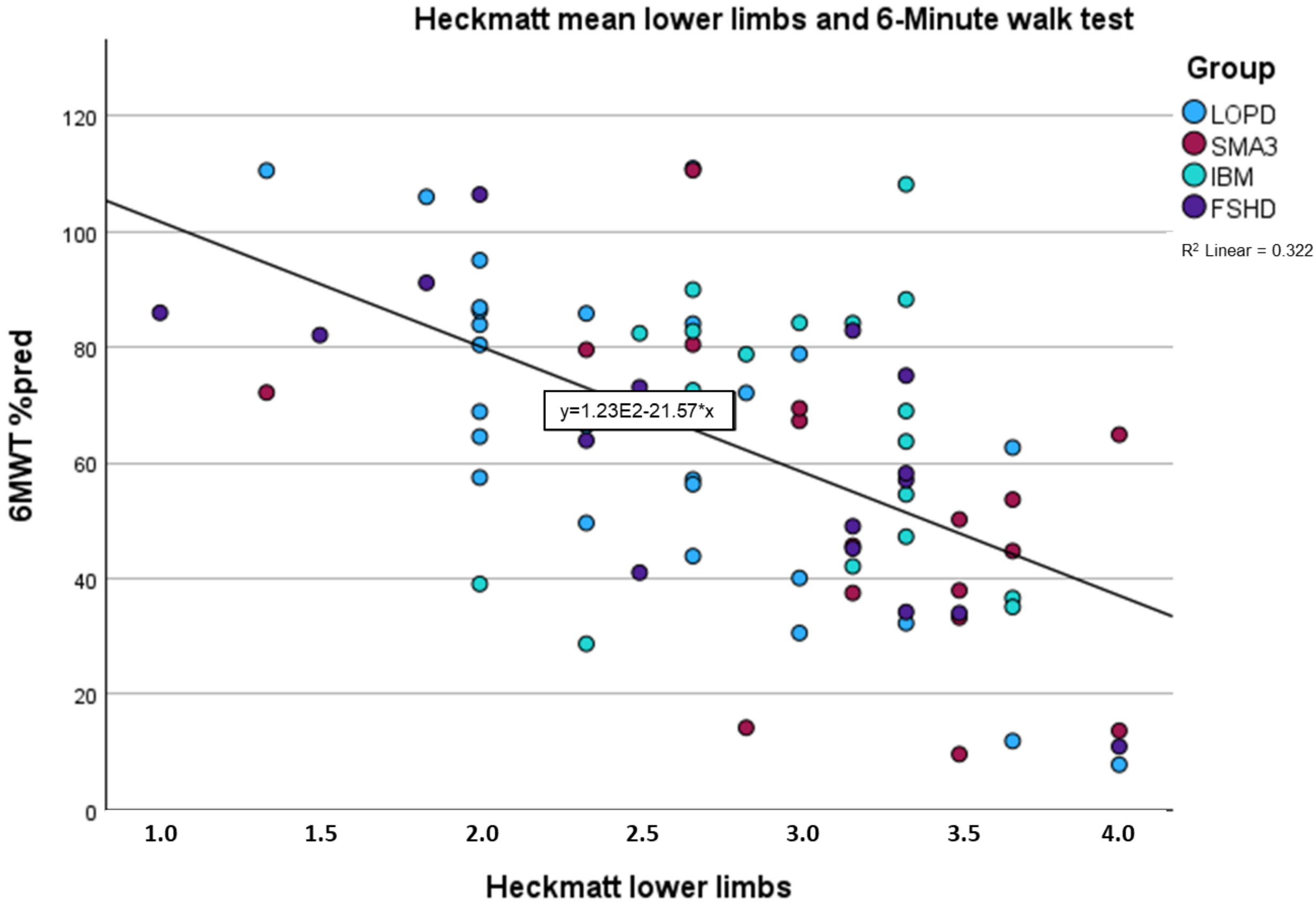

Scatterplot showing the relationship between the composite Heckmatt score of the lower extremities and the 6-minute walk test (6MWT) performance (% predicted). A significant negative correlation was observed (p=-0.574; p<0.001).
